# Percutaneous vertebroplasty in osteoporotic vertebral compression fracture with huge spinal epidural hematoma: A case report

**DOI:** 10.1097/MD.0000000000029340

**Published:** 2022-06-03

**Authors:** Huafeng Wang, Fengfei Lin, Guiqing Liang, Yuhan Lin

**Affiliations:** Department of Spine Surgery, Fuzhou Second Hospital, The Third Clinical Medical College, Fujian Medical University, Fuzhou, China.

**Keywords:** osteoporotic vertebral compression fracture, spinal epidural hematoma, percutaneous vertebroplasty

## Abstract

**Rationale:**

Osteoporotic vertebral compression fracture (OVCF) accompanying huge spinal epidural hematoma (SEH) is fairly rare. The aim of this report is to investigate the management strategies and treatment outcomes of OVCF accompanying SEH.

**Patient concerns:**

An 89-year-old female patient was admitted to hospital because of severe back pain and numbness of both lower limbs after a slight fall. The magnetic resonance imaging examination of the patient showed a fresh compression fracture at L2 accompanying a large dorsal SEH which extended from the T12 to L3 and deformed the spinal cord.

**Diagnosis:**

The patient was diagnosed with OVCF accompanying SEH.

**Interventions:**

Given mild neurologic deficits, the hematoma was not treated, and the patient underwent percutaneous vertebroplasty (PVP) only.

**Outcomes:**

After the procedure, immediate pain relief was achieved and the numbness of both lower limbs disappeared 3 days later. Three months after the procedure, the follow-up magnetic resonance imaging revealed a complete resolution of the hematoma.

**Lessons:**

OVCF accompanying SEH is fairly rare, and the exact pathophysiological mechanisms are still not clear. In selected patients without or with only slight neurologic symptoms, it is reasonable to perform PVP alone in OVCF accompanying SEH. Moreover, intravertebral stability after PVP might have played a role in spontaneous resolution of SEH.

## 1 Introduction

In recent years, percutaneous vertebroplasty (PVP) has been widely used to treat painful, osteoporotic vertebral compression fractures (OVCF).^[1,2]^ However, controversy continues to cloud the clinical role for PVP. Moreover, most patients suffering acute painful OVCF can be treated with conservative medical therapy. But in an elderly patient cohort with established osteoporosis and severe pain, many patients fail to achieve adequate pain relief and forward progress is stagnant, with serious complications and prolonged recovery.^[3,4]^

Prior studies have often excluded patients with spinal canal compromise secondary to various etiologies, including spinal epidural hematoma (SEH), from receiving PVP for fear of further spinal canal compromise.^[1]^ For OVCF with spinal canal compromise caused by retropulsion of fractured bone fragments, recent reports have suggested that PVP can be performed safely.^[5,6]^ However, there are no detailed case reports concerning the management strategies and treatment outcomes of OVCF accompanying SEH, which is a fairly rare cause of spinal canal compromise. In this paper, we assess PVP of symptomatic OVCF with SEH and to systematically review the previously reported cases in the literature.

## 2 Case presentation

**Figure 1:**
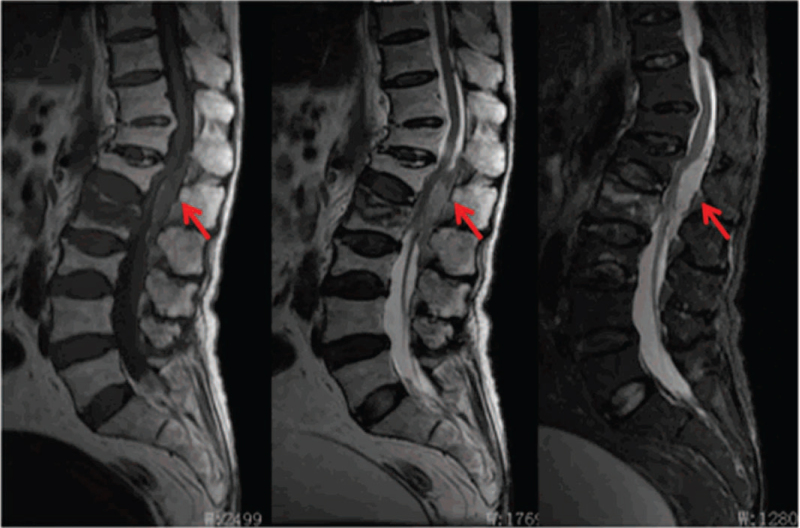
The preoperative MRI performed on admission revealed L2 fresh vertebral fractures accompanying a well-defined ovoid lesion in the dorsal epidural region at T12-L3 level that caused compression of the spinal cord. The lesion was isointense signals of the spinal cord with a thin hyperintense signal at the peripheral rim on the T1-weighted images, and mixed signal intensity on the T2-weighted images (indicated by red arrow). MRI = magnetic resonance imaging.

An 89-year-old woman presented at our emergency department after a slight fall several hours earlier. She complained of severe back pain and numbness of both lower limbs. Physical examination revealed the muscle power of 4 limbs were normal and decreased sensation to pin-prick and light touch below L1 dermatomes. Reflexes were normal. No history of anticoagulant therapy was reported. The results of coagulation studies, including prothrombin time, partial thromboplastin time, and bleeding time, were normal. The magnetic resonance imaging examination showed a fresh compression fracture at L2 accompanying a well-defined ovoid lesion in the dorsal epidural region at T12–L3 level that caused compression of the spinal cord. The lesion was isointense signals of the spinal cord with a thin hyperintense signal at the peripheral rim on the T1-weighted images, and mixed signal intensity on the T2-weighted images (Fig. 1). SEH was identified by characteristic features. There was also a chronic compression fracture at T12, L1, and L3. The patient showed severe secondary osteoporosis with a T-score on the bone marrow densitometry of −3.90.

**Figure 2:**
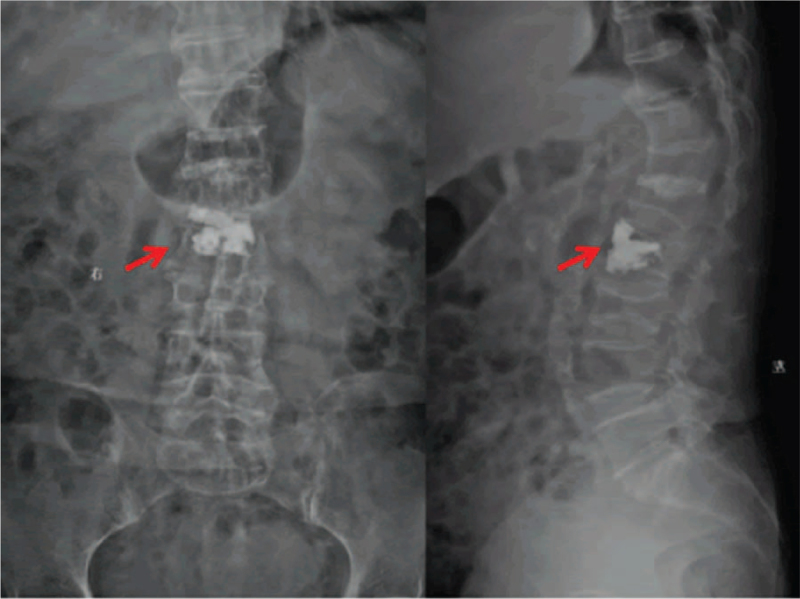
Postoperative radiographs showed that the L2 vertebral body was well filled with bone cement without intraspinal leakage (indicated by red arrow).

**Figure 3:**
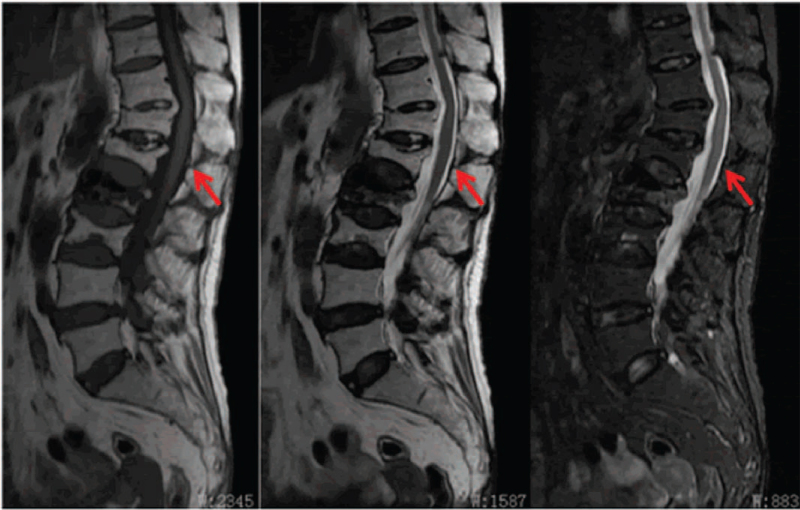
The MRI at 3-month follow-up revealed complete resolution of the spinal epidural hematoma (indicated by red arrow). MRI = magnetic resonance imaging.

Given mild neurologic deficits, no consideration was given to urgent surgical decompression and evacuation of the hematoma. After preoperative evaluation, the patient underwent PVP only under local anesthesia in prone position. The procedure was performed using standard technique.^[7]^ PVP was performed through a bipedicular approach with 11-gauge bone biopsy needles. Polymethyl methacrylate was injected under biplane fluoroscopic guidance. Care was taken that extravasation into the epidural space did not occur. After the procedure, immediate pain relief was achieved and the numbness of both lower limbs disappeared 3 days later. Postoperative radiographs showed that the L2 vertebral body was filled with cement without cement leakage into the spinal canal (Fig. 2). Three months after the procedure, the follow-up magnetic resonance imaging revealed complete resolution of the SEH and amelioration of the spinal canal compromise (Fig. 3). This study was approved by the Ethics Committee of Fuzhou Second Hospital, and written informed consent was obtained from the patient.

## 3 Discussion and conclusions

Most patients suffering acute painful OVCF can be treated with conservative medical therapy, but in an elderly patient cohort with established osteoporosis and severe pain, many patients fail to achieve adequate pain relief and forward progress is stagnant, with serious complications and prolonged recovery.^[3,4,8]^ Therefore, for elderly patient cohort with established osteoporosis and severe pain, early PVP performed may gain more clinically significant benefits.

It is often assumed that presence of spinal canal compromise is a relative contraindication for PVP, because spinal cord compression after treatment has been reported.^[1]^ For OVCF accompanying SEH, which is a fairly rare cause of spinal canal compromise, there is scarce literature concerning the management strategies and treatment outcomes. In 2006, Singh et al^[9]^ reported the first case of OVCF accompanying dorsal SEH. The authors concluded that PVP can be performed safely if the SEH was stable (the neurological symptoms were mild or normal). However, they did not describe the mechanism, treatment strategies, and prognosis of the SEH. We searched the literature in 4 electronic databases including PubMed (https://pubmed.ncbi.nlm.nih.gov/), EMBASE (https://www.embase.com), Scopus (https://www.scopus.com/home.uri), Web of Science (www.webofknowledge.com/), and ProQuest (https://www.proquest.com/) from January 1990 to December 2020, but only 4 articles on 4 cases were identified^[9–12]^ (Table 1). We report a further case of acute OVCF accompanying SEH managed successfully with simple PVP. What makes our case different from those cases in the literature, is the SEH located in the dorsal epidural space, and the fracture type is recent.

**Table 1:** Summary of reported osteoporotic compression fracture with epidural hematoma in clinical characteristics, therapeutic strategies, and treatment outcomes.

Ref.	Age, y	Gender	OVCF	Location of SEH	Neurological symptoms	Treatment	Outcomes
Present case	89	Female	L2	Dorsal; T12-L3	Numbness of both lower limbs	PVP only	Neurological symptoms improved after PVP, and complete resolution of SEH at 3 months follow-up
Singh^[9]^	81	Male	T6	Dorsal; T6 level	Normal	PVP only	Unknown
Kim^[10]^	82	Female	L1	Ventral; L1- L3	Normal	PVP only	Complete resolution of SEH at 3 days after PVP
Kang^[11]^	79	Female	T11	Dorsal; T10-L2	Progressive motor weakness and paresthesia of both lower limbs	Emergent decompressive laminectomy and PVP	Neurologic deficits improved
Hirata^[12]^	73	Female	L1	Ventral; T6 level	Unknown	PVP only	Complete resolution of SEH at 3 months follow-up

OVCF = osteoporotic vertebral compression fracture, PVP = percutaneous vertebroplasty, SEH = spinal epidural hematoma.

SEH is associated with idiopathic, iatrogenic, traumatic, and coagulation diseases, and is relatively rare in clinical practice.^[13]^ Upon review of the literature, traumatic SEH is typically associated with high energy trauma, however, there are scarce reports associated with OVCF.^[14,15]^ The exact pathophysiological mechanisms underlying OVCF accompanying SEH is still not clear. Kim et al^[10]^ and Hirata et al^[12]^ theorized that the instability of the fracture was a cause of hematoma. They suspected that the fluid including the hemorrhage inside of the intravertebral cleft may be under pressure, and be pushed out into the epidural space during daily motion, and cause a subacute or chronic SEH. However, the SEH in their cases were located anterior to the thecal sac, and the fracture type was delayed post-traumatic collapse of vertebral body (also known as the Kummell disease). Kang et al^[11]^ reported a case of delay neurological deficit induced by dorasl SEH associated with thoracic OVCF. They concluded that the SEH resulted from the hemorrhage inside the intravertebral cleft. However, in our case, the pathophysiology of SEH seemed different. Our case developed severe low back pain and numbness of both lower limbs soon after a slight fall. The SEH were located posterior to the thecal sac, and the fracture type was fresh. Although little is known, mild trauma is highly associated with SEH in the current case. The veins in the epidural venous plexus which are vulnerable to rupture are the most likely source of SEH.^[15]^

Historically, the mainstay of traumatic SEH management was urgent surgical decompression and evacuation of the hematoma.^[16]^ The prognosis of SEH appears to be related to the severity of the preoperative neurological deficits and the time to intervention; early surgical treatment is crucial for good outcomes.^[13]^ However, some cases of spontaneous recovery after traumatic SEH have been reported.^[17]^ Although the exact mechanism underlying the spontaneous resolution of SEH is largely unknown, for patients without or with only slight neurologic symptoms, conservative treatment with watchful observation appears to be a safe therapeutic option. However, little is known about the management strategies of OVCF accompanying SEH. Kang et al^[11]^ performed emergent decompressive laminectomy due to the progressive motor weakness and paresthesia of both lower limbs. Kim et al^[10]^ reported a case of chronic SEH related to Kummell disease. They suspected that the continuous instability might have prevented the spontaneous resolution of hematoma. And the stabilization after successful PVP may have contributed to the spontaneous resolution of SEH. This finding was supported by Hirata et al.^[12]^ It is interesting to note that our case regarding the location of SEH and fracture type apparently diverge from those reported in the literature. Given mild neurologic deficits and intractable back pain, the SEH was not treated, and the patient underwent PVP only. However, immediate pain relief was achieved and the neurological symptoms were resolved soon after the PVP procedure. From the experience of the authors and review of the literature, it can be speculated that if the neurological symptoms is mild or normal, PVP alone may be an effective treatment for OVCF accompanying SEH. Moreover, intravertebral stability after PVP might aid in the spontaneous resolution of the hematoma over time. Further investigations are required to confirm our findings.

In conclusion, OVCF accompanying SEH is fairly rare, and the exact pathophysiological mechanisms are still not clear. In selected patients without or with only slight neurologic symptoms, it is reasonable to perform PVP alone in OVCF accompanying SEH. Moreover, intravertebral stability after PVP might have played a role in spontaneous resolution of SEH.

## Acknowledgments

The study was supported by a grant from the “Young and Middle-aged Research Project of Fujian Provincial Health Commission of China, 2019-ZQN-86.”

## Author contributions

**Conceptualization:** Huafeng Wang, Fengfei Lin.

**Funding acquisition:** Huafeng Wang, Fengfei Lin.

**Supervision:** Fengfei Lin.

**Validation:** Guiqing Liang, Yuhan Lin.

**Writing – original draft:** Huafeng Wang.

**Writing – review & editing:** Huafeng Wang, Fengfei Lin, Guiqing Liang, Yuhan Lin.
